# Nanoindentation of 35 Virus Capsids in a Molecular Model: Relating Mechanical Properties to Structure

**DOI:** 10.1371/journal.pone.0063640

**Published:** 2013-06-13

**Authors:** Marek Cieplak, Mark O. Robbins

**Affiliations:** 1 Institute of Physics, Polish Academy of Sciences, Warsaw, Poland; 2 Department of Physics and Astronomy, Johns Hopkins University, Baltimore, Maryland, United States of America; Centro Nacional de Biotecnologia – CSIC, Spain

## Abstract

A coarse-grained model is used to study the mechanical response of 35 virus capsids of symmetries T = 1, T = 2, T = 3, pseudo T = 3, T = 4, and T = 7. The model is based on the native structure of the proteins that constitute the capsids and is described in terms of the C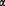 atoms associated with each amino acid. The number of these atoms ranges between 8 460 (for SPMV – satellite panicum mosaic virus) and 135 780 (for NBV – nudaureli virus). Nanoindentation by a broad AFM tip is modeled as compression between two planes: either both flat or one flat and one curved. Plots of the compressive force versus plate separation show a variety of behaviors, but in each case there is an elastic region which extends to a characteristic force 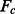. Crossing 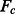 results in a drop in the force and irreversible damage. Across the 35 capsids studied, both 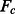 and the elastic stiffness are observed to vary by a factor of 20. The changes in mechanical properties do not correlate simply with virus size or symmetry. There is a strong connection to the mean coordination number 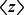, defined as the mean number of interactions to neighboring amino acids. The Young's modulus for thin shell capsids rises roughly quadratically with 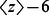, where 6 is the minimum coordination for elastic stability in three dimensions.

## Introduction

Simple globular viruses protect their strands of RNA or DNA with remarkable self-assembled proteinic shells known as capsids. The chemical and thermal stability of capsids has been studied for decades, but their mechanical properties are only beginning to be determined using nanoindentation [1], [2], [3]. There is little basic understanding of how mechanical strength varies between viruses, how it affects function, and how it is related to virus structure. In this paper we use a coarse-grained structure-based model to explore the types of mechanical response that capsids may exhibit and their relation to capsid geometry and protein bonds.

The capsids of simple globular viruses are typically of icosahedral symmetry and they are assembled from one or several kinds of proteins. The proteins cluster into 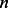-meric capsomeres that form morphological units of capsids (so the proteins act as subunits). The structure of capsids has been explained by Caspar and Klug [4] as resulting from a regular triangulation of a sphere and is thus governed by the triangulation number T such that the number of subunits is equal to 60T. A pseudo-T = 3 virus has the symmetry of a T = 3 virus, but in which either the number of subunits in a capsomere is larger than in the standard classification, or the subunits are not sequentially identical. The size of capsids tends to grow with T but the actual size also depends on the size of the subunits. One question will be whether mechanical properties depend on T or on the nature of connectivity in the network of interactions between amino acids.

Capsids are thought to be sturdy mechanically [1], [2], but the elastic properties of fewer than ten different capsids have been studied by nanoindentation [3]. For five of these capsids, the native structure is known and is deposited in the VIPERdb database [5] as derived from the subunit structures available in the Protein Data Bank [6]. These are: MVM [7]–[9] (T = 1), CCMV [10], [11] (T = 3), NV [12] (T = 3), HBV [13], [14] (T = 4), and HK97 [15] (T = 7), where the acronyms stand for parvovirus minute virus of mice, cowpea chlorotic mottle virus, Norwalk virus, human hepatitis B virus, and bacteriophage HK97 mature virus, respectively.

The nanoindentation technique typically involves anchoring a capsid on a substrate and then pushing on it with the tip of an atomic force microscope [1]–[3]. The tip typically has a larger radius than the virus and can be made nearly flat over the virus. The force, 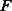, initially grows linearly as the plate/tip separation decreases. The slope corresponds to an effective spring constant, 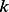. When a characteristic force, 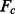, is reached, the capsid undergoes a sudden partial collapse. Beyond this point the deformation process becomes irreversible and trajectory-dependent. The values of 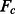 and 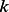 depend on whether the capsid is full or empty, i.e. if the genetic content of the virus is removed. They also depend, to a lesser extent, on the rate of squeezing. Among the five capsids listed above, 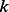 varies between 0.09 N/m for HBV and 0.57 N/m for MVM when the capsids are empty. The characteristic forces vary between 0.6 nN (CCMV) and 1.2 nN (MVM).

Here, we extend our previous theoretical studies [16] of nanoindentation of CCMV and CPMV (cowpea mosaic virus) to 33 other capsids for which the native structure is known. The capsids studied, together with their names and PDB codes, are listed in Tables 1 and 2. Also shown are the number of amino acids 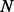, the mean radius 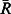, the capsid width 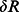, defined as twice the rms variation in radius, and the mechanical properties. Note that the values of 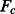 and 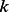 vary by more than a factor of 20.

**Table 1:** Characteristics of the T = 1, T = 2, and T = 3 virus capsids that are studied in this paper.

Acronym	PDB	common name	*N*	〈*z*〉	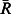 [Å]	δ*R* [Å]	*k* [ε/Å 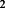 ]	*F_c_* [ε/Å]	*E* [ε/Å 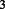 ]
**T = 1**									
MVM	1mvm	parvovirus minute virus of mice	32 940	6.76	110.54	25.51	0.217	8.7	 0.037
STMV	1a34	satellite mosaic virus	8 820	7.10	73.07	13.71	0.124	7	 0.048
FPV	1c8e	feline panleukopenia virus	32 040	7.17	109.69	25.58	0.280	13	 0.047
STNV	2buk	satellite tobacco necrosis virus	11 040	7.58	77.87	19.19	0.156	8	 0.033
IBDV	1wcd	avian infectious bursal disease virus	25 260	7.23	90.46	36.24	0.087	1.8	 0.006
SPMV	1stm	satellite panicum mosaic virus	8 460	7.34	69.66	13.28	0.174	11	 0.069
B19	1s58	B19 parvovirus	31 380	6.60	109.18	24.06	0.159	6.6	 0.030
PhiX	2bpa	bacteriophage PhiX-174	38 220	7.27	125.41	34.54	0.188	14	 0.020
PPV	1k3v	porcine parvovirus	32 520	7.14	109.40	25.14	0.291	10.5	 0.050
BmDNV	3p0s	bombyx mori densovirus 1	24 720	9.36	106.95	18.26	0.258	25	 0.082
**T = 2**									
PIC	2vf1	picobirnavirus	63 000	6.91	159.01	23.89	0.096	4	 0.027
**T = 3**									
CCMV	1cwp	cowpeak chlorotic mottle virus	28 620	6.36	119.56	21.09	0.050	5.5	 0.014
NV	1ihm	Norwalk virus	89 700	6.78	159.62	41.74	0.190	12	 0.017
RYMV	1f2n	rice yellow mottle virus	35 400	7.29	130.16	18.72	0.240	13	 0.089
CAL	2gh8	calicivirus	97 740	6.78	162.90	46.33	0.175	4.5	 0.013
TYMV	1auy	turnip yellow mosaic virus	32 460	7.24	129.35	19.89	0.224	8	 0.073
BMV	1js9	brome mosaic virus	30 180	6.40	116.91	23.19	0.053	1.5	 0.012
SBMV	4sbv	southern bean mosaic virus	37 200	7.16	131.59	18.59	0.210	12	 0.080
CMV	1f15	cucumber mosaic virus	32 280	6.48	126.27	25.06	0.056	1.6	 0.011

The first three columns show the acronym used, the PDB structure code and the common name. The next four columns give geometrical parameters from the PDB structure: the number of C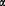 atoms describing the model capsid, their average coordination number 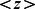 and average radius 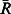 and the thickness of the capsid shell 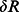 (defined as twice the rms variation in the radial direction). The final three columns give mechanical properties from simulations at 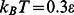: the initial spring constant 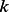, the force at the onset of irreversibility 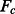, and the effective elastic modulus 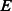 obtained using Equation 1.

**Table 2:** Similar to Table 1 but for the pseudo T = 3, T = 4, and T = 7 virus capsids.

Acronym	PDB	common name	*N*	〈*z*〉	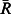 [Å]	δ*R* [Å]	*k* [ε/Å 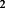 ]	*F_c_* [ε/Å]	*E* [ε/Å 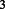 ]
**p. T = 3**									
CPMV	1ny7	cowpea mosaic virus	33 480	7.40	124.29	22.26	0.350	15	 0.088
cHRV	1k5m	human rhinovirus 16/HIV type 1V3	49 740	7.29	132.12	23.65	0.236	21	 0.056
POLIO	1asj	polio virus – type I Mahoney strain	51 060	7.52	131.64	23.70	0.500	27	 0.117
TRSV	1a6c	tobacco ringspot virus	30 780	7.19	126.86	20.870	0.188	9	 0.055
SVDV	1mqt	swine vesicular disease virus	49 860	7.33	131.70	23.14	0.474	19.5	 0.117
HRV	1ayn	human rhinovirus 16	48 240	7.51	131.60	23.50	0.443	32	 0.106
RCMV	Rcmv	red clover mottle virus	33 000	7.32	124.23	22.02	0.242	11	 0.062
MENGO	2mev	mengo encephalomyocarditis virus	48 840	7.40	133.26	21.44	0.415	24	0.120
CPV	1b35	cricket paralysis virus	51 240	7.37	135.67	23.45	0.435	19	 0.107
TME	1tme	Theiler's murine encephalomyelitis v.	46 080	7.43	133.83	20.58	0.302	24	 0.096
CVBT	1cov	coxsackievirus B3	49 860	7.63	131.66	22.76	0.543	25	 0.138
FMDV	1bbt	foot and mouth disease virus	39 720	7.32	131.40	17.84	0.250	21	 0.103
**T = 4**									
HBV	1qgt	human hepatitis B virus	34 200	6.98	145.28	22.14	0.037	6	0.011
NBV	1ohf	nudaurelia	135 780	7.49	167.66	43.60	0.438	14	0.039
**T = 7**									
HK97	1ohg	bacteriophage HK97 mature	117 600	6.76	284.03	23.18	0.08	7.5	 0.042
SV40	1sva	simian virus 40	123 420	6.95	214.40	32.34	0.058	13	0.012

cHRV denotes a chimeric human rhinovirus with a loop belonging to the (nonspeherical) HIV virus [17].

The model we use has been developed and tested in the context of single protein manipulation [18]–[22]. The model is coarse-grained, structure-based, and it comes with an implicit solvent. These features allow for studies of much larger proteinic objects than more detailed atomic models. We study capsids with up to 135 780 amino acids (NBV) and with radii up to 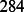 Å (HK97). Despite these simplifications, our model is molecular in nature which makes it distinct from the elastic shell model considered by Gibbons and Klug [23], [24]. While shell models with appropriate constitutive laws can reproduce the initial portion of experimental force curves, they give smoothly varying capsid deformations. They do not capture the intrinsically heterogeneous nature of the non-covalent bonds that dominate the mechanical response of capsids or allow for breaking of these bonds. Coarse-grained simulations of CCMV show deformation is localized at the boundaries of capsomeres and that 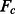 is associated with breaking of inter-protein bonds [16]. Moreover, the rate and temperature dependence of 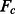 show that bond breaking is thermally activated.

Another striking prediction of the coarse-grained model is a large difference in the mechanical properties of CCMV and CPMV even though they have similar 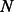, 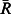 and 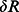. 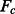 is about three times bigger for CPMV than for CCMV whereas 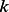 is bigger by an order of magnitude [16]. This would require very different elastic properties to be used in an elastic shell model. We found that the difference correlated with differences in the average coordination number in the native state 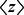, including connections along the backbone and to nearby amino acids. The average coordination number is only 6.4 for CCMV and rises to 7.4 for CPMV. Here, by examining a wider variety of capsids, we are able to show that 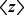 is indeed an important factor in determining the maximum stiffness of capsids of a given 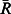 and 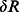. The results for 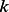 can be scaled to give an effective Young's modulus 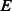 using the formula for thin shells. This local property shows a quadratic trend with 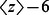, where 6 is the minimum coordination number for mechanical stability in three dimensions.

In the following section we describe the model used for the simulations. We then present results for CCMV to illustrate the role of temperature and a finite radius, 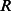, of the tip. A finite 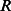 has little effect on 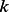 but reduces 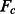. Next, results for five systems are compared with existing experimental data. The model captures quantitative trends and shows capsids can exhibit a variety of qualitatively different force-distance curves. The next section compares results for all 35 virus capsids and the final section presents a summary and conclusions.

## Methods

We start by presenting the pertinent aspects of the model. The molecular dynamics [25] model we use is exactly the same as in ref. [16]. In particular, native contacts between C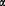 atoms are identified based on the overlap of the (slightly enlarged) van der Waals spheres associated with the heavy atoms of the amino acids [26]. Only capsids whose native state is available [27], [5] are considered. It should be noted that in most cases the positions of some amino acids are not determined. These are not part of the fixed capsid structure and do not scatter coherently. These segments are believed to dangle inside the capsid in a disordered configuration and may be important for encapsulating RNA or DNA [28], [29]. Since they do not stay in fixed positions, we assume that they do not contribute to the mechanical stability of the capsid.

The interaction for the native contact between atoms 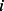 and 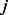 at distance 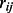 is described by the Lennard-Jones potential 
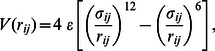
 where 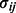 is determined for each pair 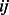 so that the potential minimum coincides with the experimentally determined native distance. A native contact is considered broken if 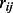 exceeds 1.5 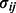. Interactions between atoms that are not part of a native contact are purely repulsive and are given by a Lennard-Jones potential with length 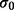 that is truncated at the position of the energy minimum 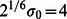
Å. Covalent couplings along the protein backbones are described with a harmonic potential with spring constant 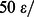
Å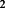. This value is high enough that covalent bonds are effectively rigid during capsid deformation but small enough that the equations of motion can be integrated without reducing the time step significantly.

Simulations are performed at a constant temperature using a Langevin thermostat [30] that mimics the effect of the surrounding solvent. The Langevin damping is large enough that the system is in the overdamped limit where inertia can be ignored. Based on mapping simulations to experimental measurements of dynamic quantities such as diffusion, the time unit 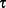 in our simulation corresponds to 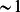
ns [31], [32]. Except as noted, simulations are at temperature 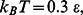 which was used in previous studies of capsids [16] and many mechanical studies of proteins [22], [18], [19]. At this temperature, most of the proteins have good folding properties within the coarse-grained model [18]. In comparing to experiments we will use our most recent estimate of the binding energy parameter 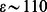 pN/Å, which is based on comparison to forces from experiments on protein stretching [22]. This energy corresponds to about 800 K, which would imply room temperature is closer to 0.35 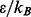 than 0.3 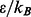. This difference is comparable to the uncertainty in the estimate of 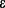 and we will show that mechanical properties generally vary slowly with temperature.

To model nanoindentation the capsid is placed between two repulsive plates. The interaction with flat plates is described by a repulsive potential that scales as 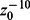 [33], where 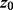 is the distance between the plate and a C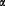 atom. A curved plate is described by the truncated and shifted Lennard-Jones potential 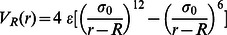 for 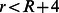
Å. A typical value of 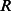 is 30 nm. Whenever we refer to the “curved” case of nanoindentation, we mean a situation in which one plate is flat and the other is curved. Flat plates are oriented normal to the 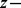 axis and capsids are oriented so that this axis coincides with the 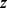-axis in the structure file [27], [5]. This is the 2-fold icosahedral axis. We have considered two additional sqeezing directions for CCMV and CPMV in ref. [16]. There was some change in force-separation curves with capsid orientation, but the values of 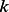 and 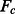 did not change significantly.

In the initial state, the plates are far enough apart that they do not interact with the capsid. The plates are then brought together by increasing the speed of both plates symmetrically to a value 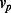 over 2000 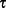. In most cases considered here, 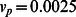 Å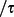 which corresponds to a combined speed of 0.005 Å/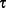. This was found to be slow enough to produce quasistatic results in studies of protein stretching [20], [21]. While this velocity (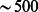 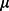
m/s) is higher than experimental velocities (0.1 to 10 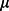
m/s), it is slow enough to allow stress to equilibrate across the capsid. This is monitored by checking that the forces the capsid exerts on the two plates are equal and opposite when averaged over a small range of separations. The magnitudes of these forces are averaged to obtain the total compressive force, 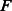, that would be measured by an AFM. This force is studied as a function of the separation 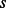 between the plates. For the case of a curved surface the separation is measured between the closest points.

## Results

### Mechanostability of CCMV – dependence on the temperature and on the curvature of the plate

**Figure 1:**
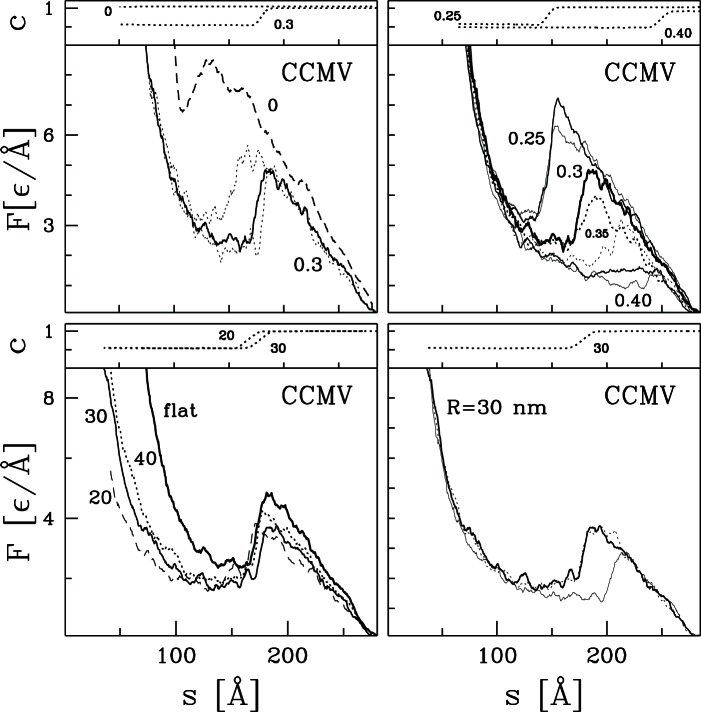
Nanoindentation of model for CCMV. The figure contains four panels, each consisting of two subpanels. The lower subpanel shows the variation of force with separation and the upper shows 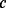 – the fraction of unbroken native contacts for one simulation at the indicated parameters. The top two panels show the temperature dependence of 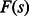 for flat plates, with numbers indicating the value of 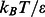. The left shows three trajectories for 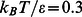 from Ref. [16] and one for zero temperature that is uniformly higher. The right repeats one 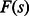 trajectory for 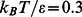 and shows two trajectories for each other temperature. The value of 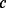 is plotted for one case at the high and low temperatures. The two lower panels show the role of curvature in one of the indenting surfaces. Here, 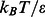 = 0.3 and numbers indicate the radius of the tip 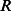 in nm. The lower left panel compares single trajectories obtained for three values of 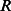 to the flat results above. The lower right panel shows three trajectories for 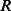 = 30 nm.

We now illustrate the type and magnitude of changes produced by temperature and 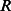 by considering the CCMV capsid. Figure 1 shows examples of nanoindentation trajectories for this capsid under various conditions. Note that experimental data are typically plotted vs. the displacement of the tip towards the substrate, which becomes more positive as 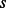 decreases. In addition, raw AFM data needs to be corrected for the compliance of the cantilever, while there is no compliance in our simulations.

In the top left panel there are three trajectories 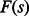 obtained at 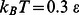 that are reproduced from ref. [16]. They, and especially the central highlighted one, provide a reference for all of the results presented here. The three trajectories nearly coincide at the beginning of the sqeezing process. In particular, there is no force for 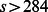 Å. The force then rises linearly with slope 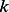 to a characteristic force 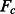 as 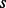 decreases to about 175 Å. This corresponds to compression by about 35% from the force onset which is comparable to what is observed in experiments on CCMV [10], [11]. The value of 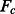 is of order 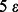/Å, i.e. about 550 pN, which is also close to the experimental value of 600 pN [10], [11]. In the linear regime, the indentation process depends only weakly on the squeezing speed and is reversible: reversing the velocity of the plates results in approximate retracing of the 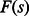 curves. When the separation is reduced beyond the linear regime, there is a sudden drop in the force and then a steep rise at small separations due to steric repulsion. Once the force has dropped, the deformation is not reversible. If the plates are retracted, the force falls rapidly to zero [16].

The onset of irreversibility in our simulations of CCMV at 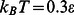 is associated with rupture of native contacts [16], rather than a buckling transition like that observed in elastic shell models [24], [10], [23]. The top subpanel of the top left panel in Figure 1 shows the fraction, 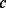, of unbroken native contacts as a function of 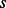. In the initial stage of compression, the number of unbroken contacts remains essentially equal to that in the native structure, although thermal fluctuations are strong enough for a few contacts to fluctuate between broken and unbroken states at 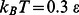. At the end of the linear regime, nearly 50% of the contacts rupture as the force drops rapidly. Most of these bonds connect different proteins within the capsid, rather than different parts of the same protein [16]. These bonds do not reform on the time scale of our simulations, but may reform in experiments on the time scale of many minutes [3].

Several aspects of the results show that the bond rupture at 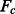 is thermally activated. The top right panel of Figure 1 shows the temperature dependence of 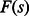. For all 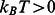, the value of 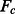 depends on trajectory. While the stiffness decreases only weakly as 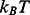 increases, 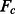 drops significantly and is almost completely suppressed at 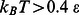 for CCMV. These results are consistent with thermal fluctuations being able to activate the transition at lower 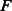 as 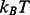 increases and to produce run-to-run variations in 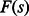 trajectories. Our previous studies showed that reducing the speed of compression, and thus reducing the time for activation, raises 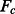 [16].

For all nonzero temperatures shown, we find a sharp drop in 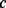 as the force drops below 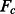. However at 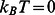 there is a drop in 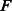 that is not associated with bond breaking and occurs at very small separations. Since there are no thermal fluctuations, the drop occurs at a well-defined separation. This case appears to reflect a buckling instability of the capsid like that predicted for elastic shell models [24], [10], [23].

We now consider the influence of tip curvature on capsid response. The lower left panel of Figure 1 compares the highlighted trajectory obtained for the case of flat walls to examples of trajectories obtained when one of the pushing walls is curved. Three values of 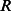 are considered: 40, 30, and 20 nm. The lower right panel shows examples of three trajectories obtained for 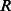 = 30 nm – a value which is typical for AFM-based nanoindentation. We observe that the sharper the tip, the easier the destruction of the capsid: both 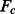 and 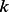 get reduced because the tip force is focused on a smaller number of proteins. Nevertheless, the flat wall results remain good estimates of what would be measured by employing, say, a 30 nm AFM tip.

### Nanoindentation for capsids considered experimentally

**Figure 2:**
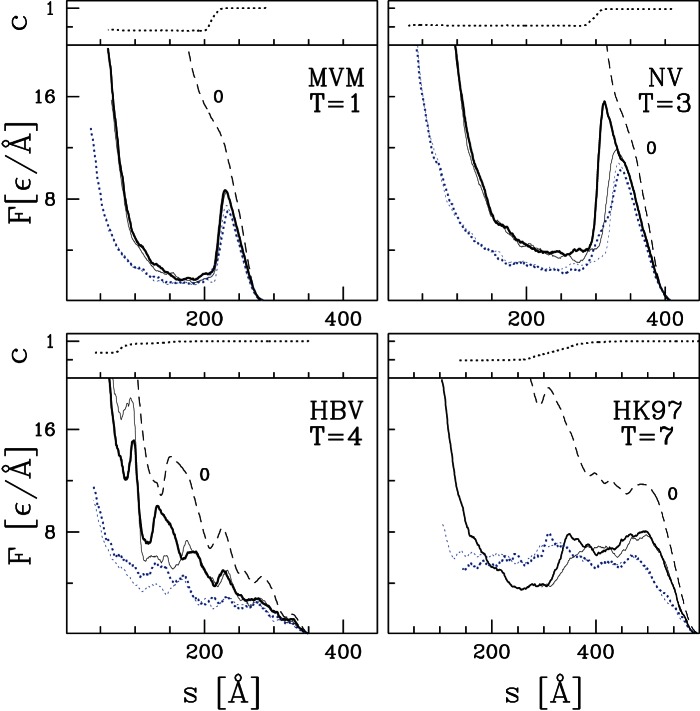
Nanoindentation processes in models for four experimentally studied capsids MVM, NV, HBV, and HK97. As in Figure 1, each panel consists of two subpanels. The lower subpanel shows several 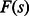 curves and the upper subpanel shows 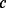 for the highlighted trajectory. For each capsid, two trajectories are shown for flat plates (solid lines) and for a tip with 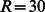
nm (dotted lines) at 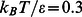 and one trajectory is shown for flat plates at zero temperature (dashed line).

As mentioned in the Introduction, nanoindentation measurements have also been made for MVM, NV, HBV, and HK97. Figure 2 shows 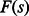 traces for coarse-grained models of these capsids at 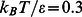 and 0 with flat and curved walls. All lines show a rapid upward curvature in the first 5–10 Å after contact. In this range the repulsive potentials from the confining walls are just beginning to overlap with the outer atoms and it is not included in fitting the spring constant 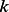. At smaller separations the traces illustrate the four types of behavior found for the full range of capsids studied later. These are a single peak at 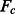 (MVM), a peak with a shoulder (NV), a series of gradually rising peaks before 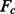 (HBV), and a peak followed by a long plateau (HK97). Note that curved walls do not change the type of force trace. As seen in the previous section, there is just a slight reduction in 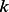 and 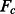 because the compressive stress is focussed on a smaller region. The magnitude of this shift is largest and most consistent between different runs for HK97 and HBV.

The traces for MVM and NV are relatively simple. At 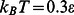 there is a single main peak followed by a sharp drop where about half the native contacts break. The main difference is that some traces for NV show a shoulder before 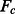 and others break at the same separation (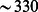
Å). At 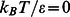, the force peak is replaced by an inflection point and no bonds break. This indicates that the capsids are always mechanically stable against small perturbations, but can fail through thermally activated bond breaking at high enough temperatures and long enough times.

A series of peaks is superimposed on a steadily increasing force in the case of HBV. This complicates the determination of 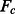. For 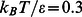, the majority of bond breaking occurs after the final peak at about 15 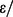
Å. A sufficient number break near 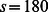
Å and 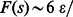
Å, to hinder reversibility and we take this as 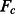. In analyzing experimental data, the first sharp peak at 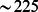 Å and 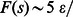 Å might be reported as 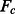 even if retraction would have produced a relatively reproducible force trace. Thus there are greater systematic uncertainties in 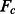 for capsids in Tables 1 and 2 that exhibit this type of force trace. The force trace at zero temperature shows peaks at very similar 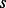 with no bond breaking. This indicates that the capsid loses mechanical instability even in the absence of thermally activated bond breaking and the nature of these instabilities will be the focus of future studies.

Finally, in the case of HK97, there is a weakly articulated force peak followed by a plateau. The plateau ends with a drop for 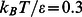 and flat walls, and extends to the onset of steric repulsion for curved walls and at zero temperature. Significant bond breaking does not occur till 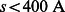 which is far along the plateau. In addition, the first peak occurs near 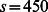
Å for all temperatures. Once again, this is indicative of a mechanical instability like the buckling instability seen for thin shells. Note that 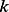 is particularly difficult to determine for HK97. The initial slope for forces up to about 2
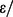
Å is used in the table. At lower separations the flat wall results rise more steeply, while the tip results do not. Results for HK97 may be more sensitive to tip size than other capsids because it is the largest and has a radius that is more than double most other capsids. As the force grows, the tip may push through the capsid like a needle. A more extreme case was considered in Ref. [34] where the effective tip radius was only a few Å and it passed completely through the capsid shell.

**Figure 3:**
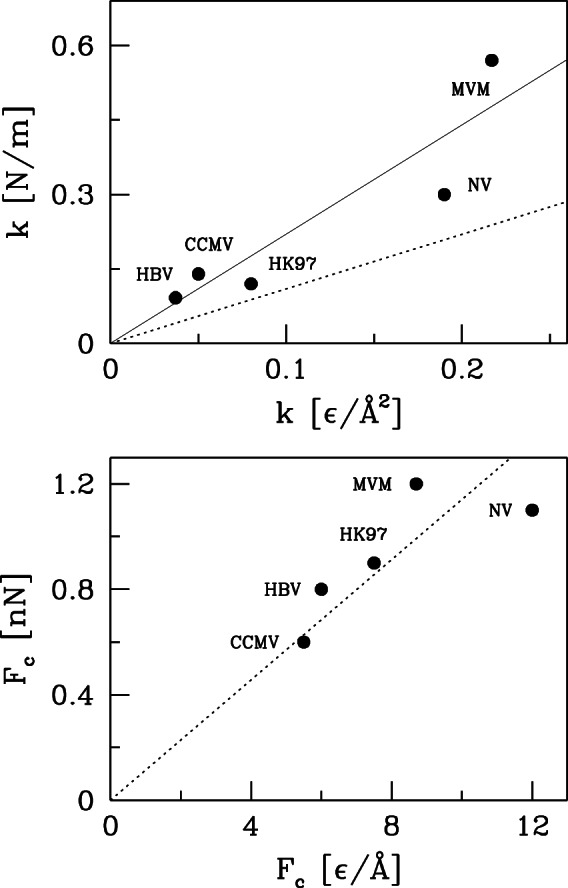
Comparison of experimental and theoretical results for 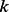 (the top panel) and 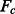 (the bottom panel) for the five indicated capsids. The dotted line is obtained using the value 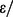
Å
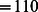
pN obtained by fitting mechanical stretching of proteins [22]. Results for 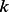 are better fit if this quantity is doubled (solid line).

Figure 3 compares theoretical results for 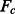 and 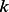 with experimental findings. Note the clear linear correlation between theoretical and experimental values. Indeed, using the value of 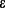 determined from matching forces from the model to protein stretching experiments [22] gives forces that are quantitatively similar to capsid experiments (dotted lines). Note that a variety of force curves are obtained for MVM. The highest and most pronounced peak in Fig. 2c of ref. [9] is at 1.2 nN, which is consistent with the trend shown in the lower panel of Figure 3. Some capsids showed no instablities up to this load, while others showed precursor peaks. These variations and observations of capsid geometry. indicate that capsid orientation and thermal activation of bond breaking are important and that failure is localized between proteins making up different trimers. Failure of interprotein bonds is consistent with our earlier results on CCMV [16].

Experimental values of 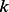 are better fit by increasing the correspondng theoretical values by a factor of 2 (solid line). As explained in Ref. [16], the only length scale included in the coarse-grained model is the separation between C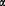 atoms in native contacts and this determines both the structure and the rate of change of forces with separation. The actual rate of change of forces will be determined by the shorter distances separating individual atoms of the amino acids associated with each C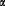. This will increase 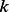 relative to the coarse-grained prediction as seen in Fig. 3. We conclude that the coarse-grained model can be used to predict trends in mechanostability for empty virus capsids and provide quantitative estimates for 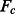 using 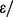
Å = 110 pN while 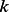 should be doubled for this normalization.

### Nanoindentation processes in other virus capsids

**Figure 4:**
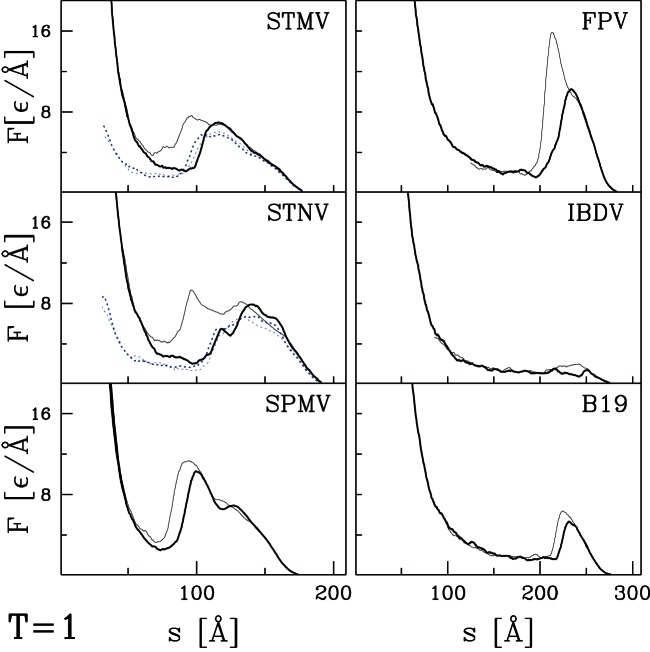
Plots of 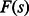 at 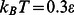 for six capsids of the T = 1 symmetry (or T = 1) studied in this paper. The solid lines are different trajectories for two flat indenting planes. The dotted lines, if any, correspond to the case where one surface has radius of curvature 30 nm.

**Figure 5:**
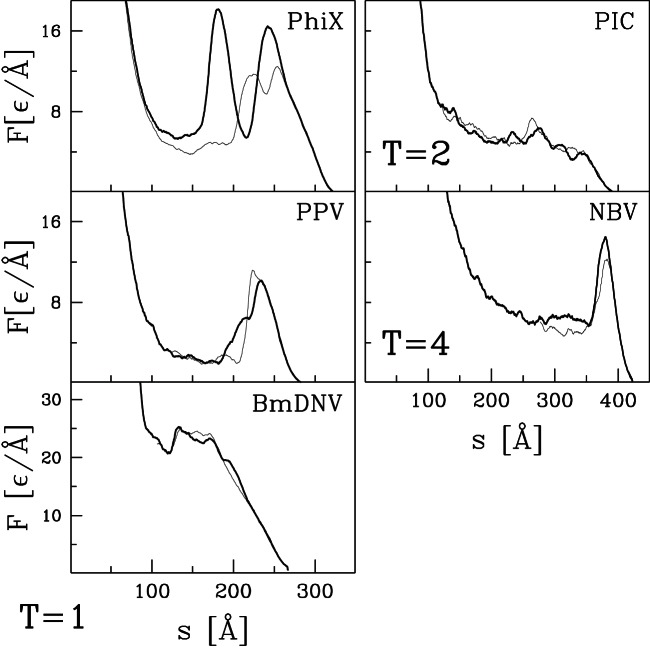
Similar to Figure 4 but for three remaining T = 1 capsids, one T = 2, and one T = 4.

**Figure 6:**
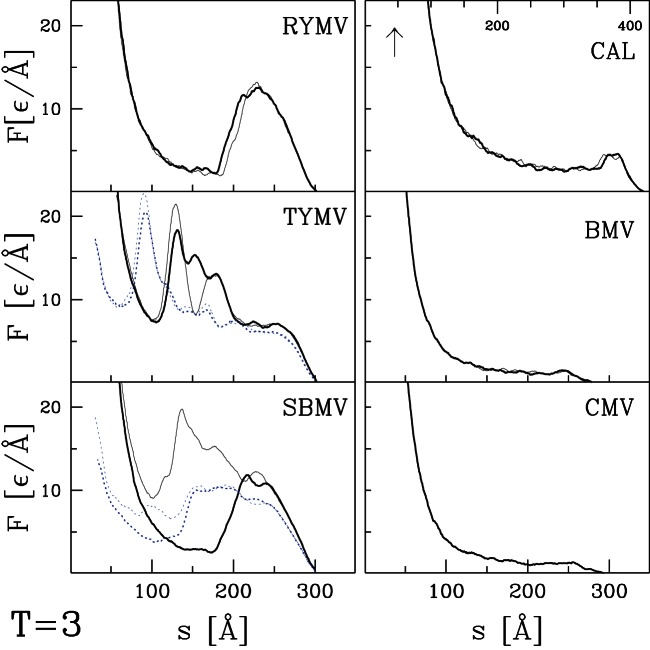
Similar to Figure 4 but for six T = 3 capsids listed in Table 1.

**Figure 7:**
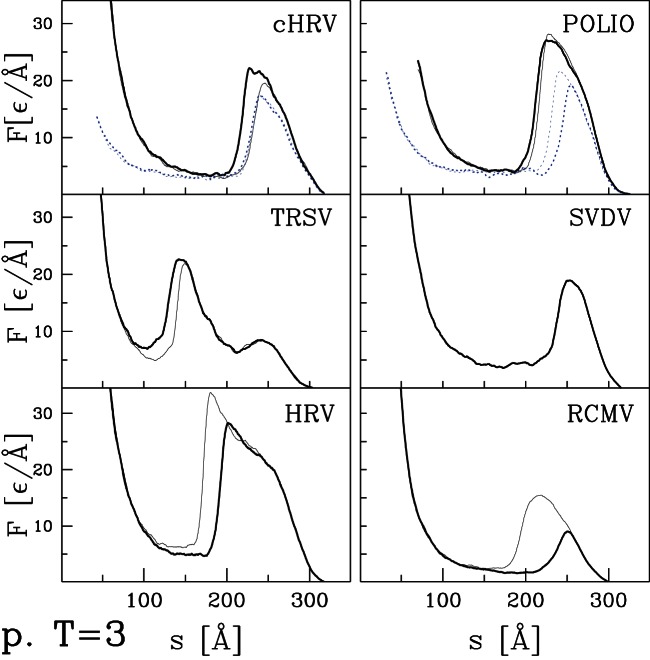
Similar to Figure 4 but for six pseudo T = 3 capsids listed in Table 2.

**Figure 8:**
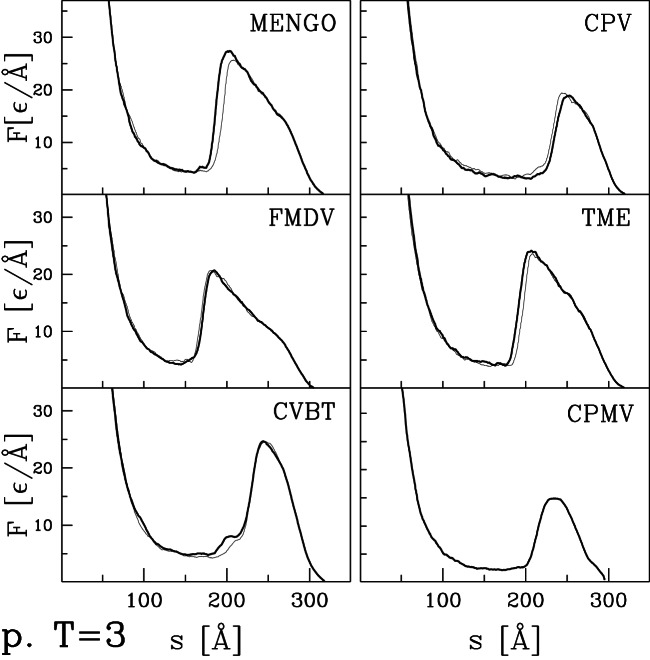
Continuation of Figure 7 for the six remaining pseudo T = 3 capsids listed in Table 2.

**Figure 9:**
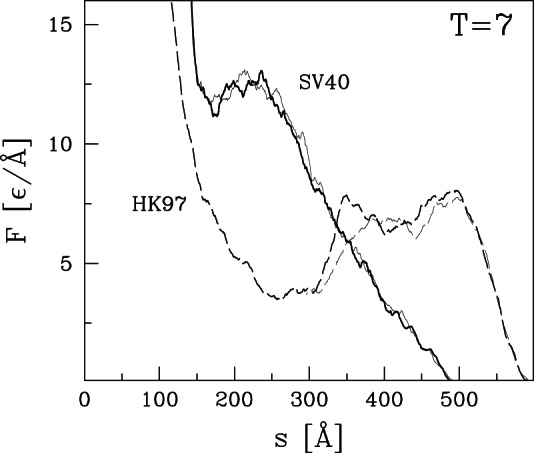
Plots of 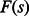 for two capsids of the T = 7 symmetry studied in this paper. The indentation is implementd by flat walls. The solid lines are for SV40. The dashed lines are for HK97 – these lines are taken from Figure 2 to make a comparison.

We now present the 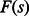 curves for the remaining capsids listed in Tables 1 and 2. Their selection was based entirely on availability of structural information and they can be viewed as an essentially random sample. Figures 4 and 5 present results for capsids belonging to the T = 1, T = 2, and T = 4 structural classes. Figure 6 corresponds to the T = 3 class, Figures 7 and 8 to the pseudo T = 3 class, and Figure 9 to the T = 7 class. Most of the results have been obtained with flat walls and are shown by solid lines.

For some capsids, dotted lines show results for a tip with radius of curvature 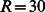 nm. As in the previous section, the curved tip does not change the type of force curve and produces a small decrease in mechanical strength. In most cases there is little change in 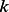. The exception is HK97 (Fig. 2) where 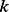 drops about 15%. Changes in 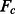 tend to be larger than those in 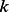, but the largest change is a 25% drop for POLIO (polio virus – type I Mahoney strain). Given the large number of viruses considered, we expect that these results provide reasonable bounds for the magnitude of changes in mechanical properties that would be produced by changing the radius of an AFM tip from nearly flat to a typical value of 30 nm.

In most cases, the 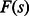 curves show a well defined single force peak that is not very sensitive to the choice of trajectory. Several show a change in slope before the peak and in a few cases this developes into a weak peak: STNV (T = 1), SPMV (T = 1), BmDNV (T = 1), and SBMV (T = 3). More and larger peaks are seen for PhiX (T = 1), PIC (T = 2), TYMV (T = 3), and TRSV (pseudo T = 3). A few force curves show a weak peak followed by a low plateau: IBDV (T = 1), CAL (T = 3), BMV (T = 3) and CMV (T = 3). The different types of behavior are not uniquely associated with specific values of T, but weak plateaus are more common for T = 3 capsids and all but one pseudo T = 3 capsid exhibits a single sharp peak. There is also no clear correlation with capsid size. Capsids with weak peaks and plateaus tend to have larger values of 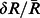 than other capsids in the same structure class, but NV has a sharp peak and a thick capsid.

The structure in 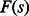 and variability between trajectories leads to systematic uncertainties in determining 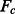. When there is a single major peak or clear plateau, we take the average height over trajectories, including some that are not shown in the figures. When there is a shoulder where some trajectories show sharp force drops, we use the shoulder height. The choice is less clear for cases with multiple peaks and leads to significant uncertainty for HBV (T = 4), TYMV (T = 3), and TSRV (pseudo T = 3). The quoted values correspond to the height of the lowest significant peak, while later peaks are a factor of two higher. The highest value is for HRV with 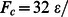
Å corresponding to about 4 nN. The smallest values for BMV, CMV and IBDV are about 20 times smaller.

**Figure 10:**
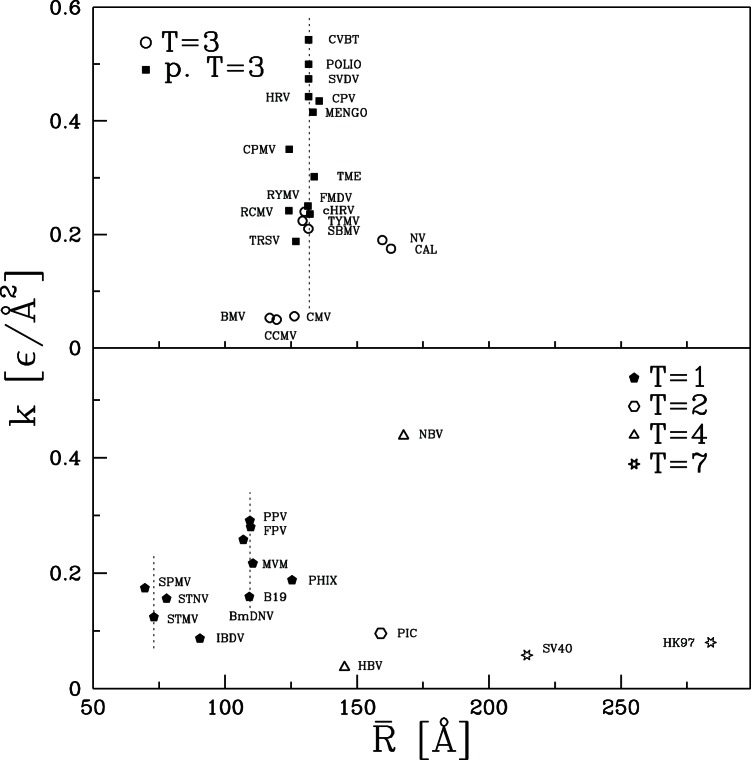
Simulation values for the effective spring constant plotted against the average radius of the corresponding capsid. The top panel is for capsids of symmetry T = 3 and pseudo T = 3 and the bottom panel for the remaining capsids. The vertical dotted line in the top panel indicates 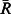 of 132 Å. In the bottom panel, the two similar lines correspond to 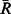 of 73.0 and 109.4 Å.

**Figure 11:**
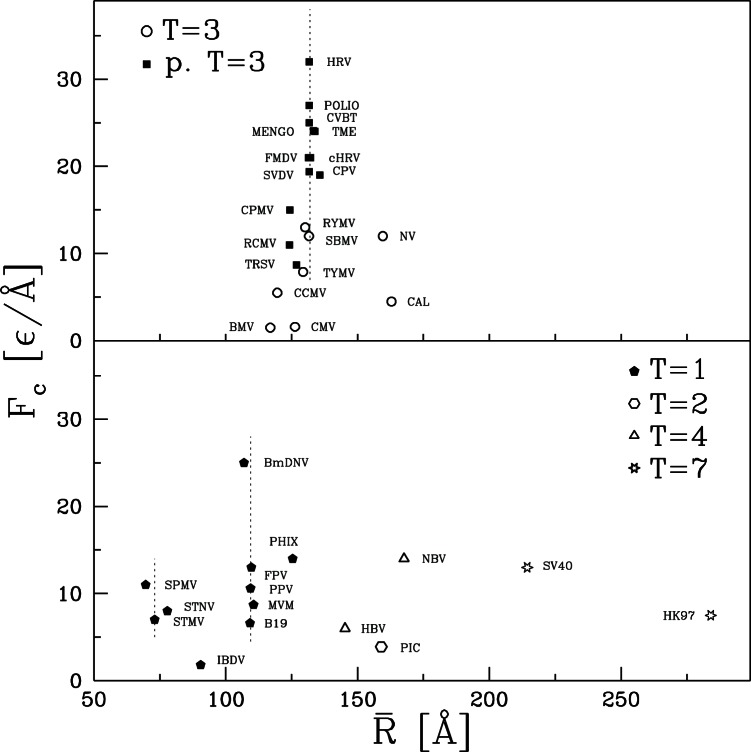
Similar to Figure 10 but for the values of 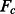.

We now ask: what do the values of 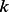 and 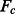 depend on? A natural attribute of the capsids to consider is their size as characterized by 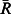. The size dependence of 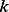 and 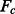 is shown in Figures 10 and 11, respectively. There is clearly no simple relation of mechanical properties to 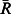. The largest capsid, HK97, has half the stiffness of the smallest capsid, SPMV, but bigger variations in 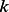 are found between capsids with the same radius. The vertical dashed lines indicate values of 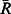 where there are many viruses of nearly the same size. Twelve of the T = 3 and pseudo T = 3 capsids have radii within 4 Å of 132 Å and four more are less than 8 Å smaller. Despite their similar size, this group contains the largest and smallest values of 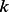, with values varying by a factor of 16 from 0.034 to 0.543
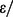
Å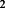. Values of 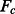 vary by a factor of 20. The cluster of five T = 1 viruses within 2Å of 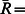 108 Å (B19, MVM, FPV, PPV, BmDNV) also shows wide variability.

Another important factor might be the structural classification. The pseudo T = 3 capsids tend to be strongest. The weakest are T = 2, T = 7 and some of the T = 3 capsids. T = 4 capsids span the full range of strength, while T = 1 and T = 3 span intermediate values. These observations indicate that there is some correlation to structure, but not a strong one.

In our previous study of CCMV and CPMV we noted that while most of their geometrical properties were very similar they had very different average coordination numbers 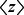. When counting non-bonding contacts and the two covalently bound neighbors along the backbone, 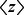 is 6.36 and 7.40 for CCMV and CPMV, respectively. The minimum number of neighbors required for stability in 3 dimensions is 6 if, as here, there are no frictional or bond angle forces. Studies of rigidity percolation indicate that the elastic modulus is a strong function of 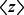 near the onset of rigidity [35]–[38]. We argued that this could explain why increasing the number of native contacts per atom by only 45% (and total bonds per atom by 30%) could lead to an order of magnitude increase in stiffness for CPMV relative to CCMV.

**Figure 12:**
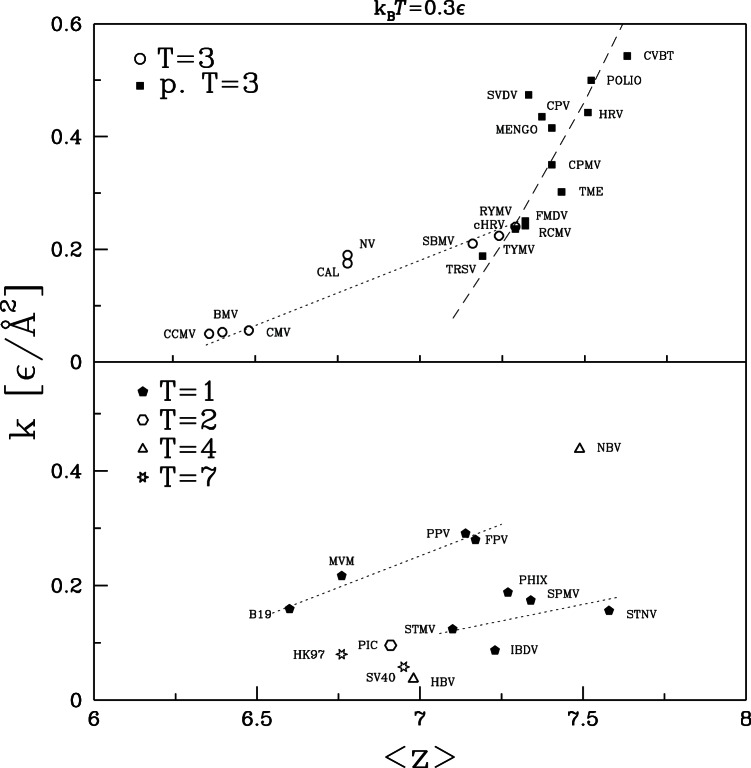
Effective spring constant plotted against the average coordination number of the corresponding capsid. The top panel is for capsids of symmetry T = 3 and pseudo T = 3 and the bottom panel for the remaining capsids. Lines in the top panel show trends for T = 3 (dotted line) and pseudo T = 3 (dashed line). In the lower panel, the two lines link capsids with 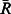 close to 73.0 and 109.4 Å respectively.

**Figure 13:**
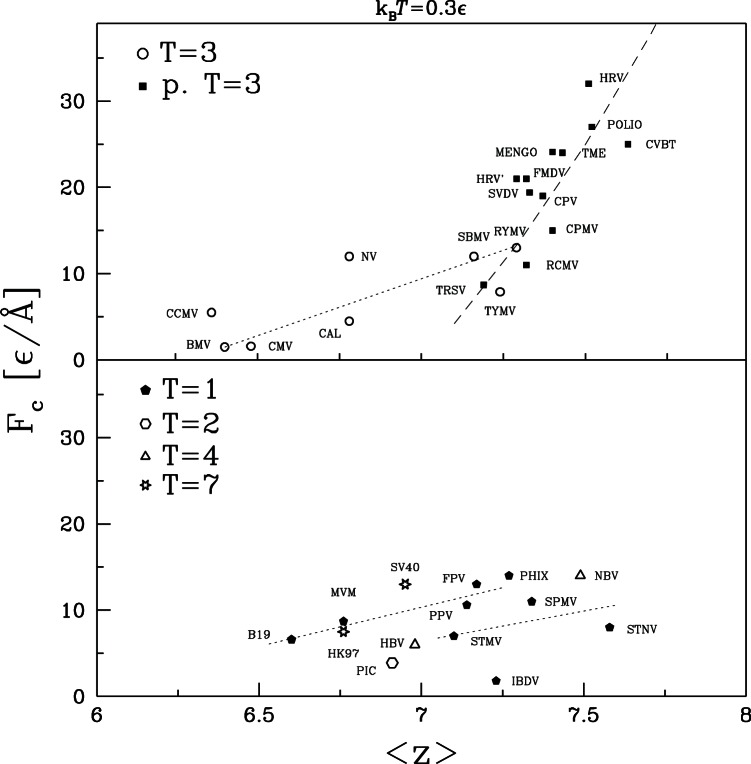
Similar to Figure 12 but for the characteristic force.

To explore the effect of coordination number, the results for 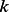 and 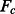 are replotted against 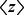 in Figures 12 and 13, respectively. For the T = 3 and pseudo T = 3 capsids there is a clear tendency for 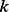 and 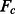 to grow with increasing 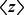. There is no clear trend for the other capsids when viewed as a group. However, they have a much larger range of sizes than T = 3 and pseudo T = 3. The dotted lines in the lower panel show that there is a clear trend for mechanical strength to grow with 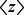 if capsids with similar radii are considered separately.

To try to separate the effect of capsid dimensions from the local modulus describing the mechanical properties within the shell, we follow a dimensional analysis motivated by the elastic shell models of Gibbons and Klug [23]. In the thin shell limit, the stiffness is proportional to the Young's modulus 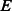 and the square of the shell thickness and inversely proportional to the radius. Up to a numerical prefactor that would depend on how thickness is defined, one can use this scaling to define an effective modulus.
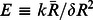
(1)


that characterizes the local response in the shell (Tables 1 and 2).

**Figure 14:**
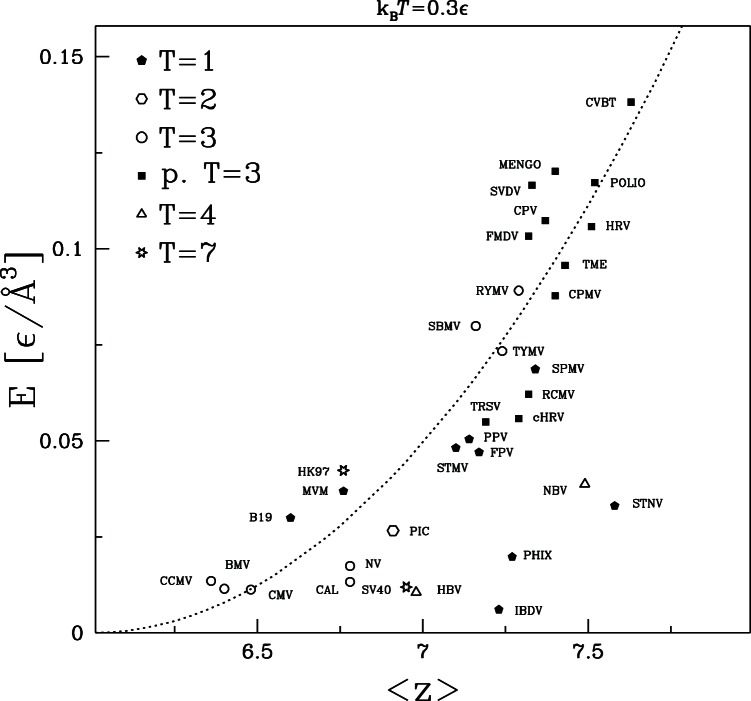
Similar to Figures 12 and 13 but for the Young modulus. The parabolic fit corresponds to the formula 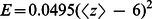 [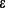/Å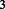].

Figure 14 shows how 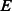 varies with 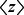 for all capsids studied. There is a much clearer correlation between these quantities than found in the previous plots. The majority of the capsids show a roughly parabolic dependence on the excess above the minimum coordination number for rigidity.
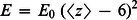
(2)where 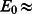 0.05 [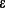/Å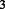]. This correlation is quite good given that there is no correction for the fact that 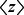 will be lower for the significant fraction of C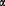 that lie on the outer and inner surfaces of the capsid, and that the distribution of bonds may be nonuniform. For example, reduced local coordination along the boundaries between capsomers could greatly lower the global stiffness 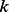 and explain why bond breaking may localize there [16]. It is also interesting to note that the greatest outliers (IBDV, PhiX, NBV and STNV) are those that are farthest from the thin shell limit, having 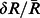 greater than 0.24. The thin shell formula is questionable in this limit and the loss of coordination number due to surfaces would be smaller than for other capsids.

Studies of randomly linked systems have also found a power law relation between modulus and the excess coordination. Square and Kagome lattices are marginally stable when only nearest-neighbors interact. Their shear modulus grows as the square of the number of next-nearest-neighbor bonds [37], which is consistent with Fig. 14. A different linear scaling is found for completely random networks near the jamming transition [38]. Capsids may be closer to the orderly structure of lattices because of the backbone connectivity and repeated structure, but it is not clear that they should fit into either class.

It should be noted that the coordination number for a given capsid is sensitive to the details of the determination of the contact map. Changing the details would alter the number of native contacts and thus 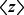. However, we expect that the trend for local elastic properties to rise rapidly with 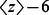 would remain. The overlap criterion used is based on previous studies of protein folding and stretching, where it has been shown to provide reasonable results [20], [22].

## Discussion

A coarse-grained molecular model was used to study the mechanical response of 35 virus capsids. The full force-separation curves have a variety of shapes, but in general share two common features. In particular, there is a linear elastic response characterized by a spring constant 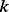 at small deformations and a sharp drop or plateau at a characteristic force 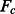 that signals an irreversible instability. As found previously for CCMV [16], 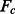 is usually associated with bond breaking at finite temperature. Because bond-breaking is thermally activated, there are run-to-run fluctuations in 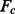, and 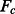 decreases with increasing temperature and increases with increasing indentation rate. Similar effects have been seen in previous simulations of mechanical unfolding of proteins where our model captures the breaking of bonds during experiments on unfolding. There is rarely any bond-breaking in capsids at zero temperature, indicating that the bonds are always metastable. Some capsids show sharp instabilities without bond breaking at zero temperature and large forces that is indicative of a buckling instability like that seen in elastic shell models [23], [24].

The elastic response and onset of irreversibility have also been considered by Arkhipov et al. [39], [40] using an even more coarse-grained model based on capsid geometry where a single bead represents 150 protein atoms, on average. Bonds are defined based on proximity of these larger beads and bond-angle terms are introduced to include the effect on rigidity of the many additional atoms that have been removed. All interactions are then fit to atomistic simulations of capsids. No bond-breaking is possible in this model because the bonds are strictly harmonic, but capsids undergo instabilities like those seen here at zero temperature. The model has been used to study the native state stability of several T = 1 and T = 3 capsids (STMV, SPMV, STNV, BMV) [39] and to get the pre-collapse behavior of the force-indentation curves for the T = 4 capsid HBV [40]. It is interesting that even with the greater coarse-graining, their results for HBV show a sinuous character that is similar to our results.

For the capsids studied in this paper, the values of 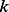 and 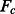 vary by about a factor of 20. These variations are not correlated with virus symmetry (T) or size. Indeed, nearly the full range of values is sampled by T = 3 and pseudo T = 3 capsids with radii between 130 and 134Å. The greatest correlation was found with the coordination number 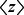 that describes the number of bonds constraining motion. To isolate the effective local elasticity from geometrical effects, we determined an effective Young's modulus 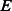 for regions within the capsid shell using the thin elastic shell formula for stiffness (Eq. 1). On average there is a trend for 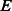 to rise quadratically with 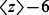, where 6 is the minimum coordination for stability in three dimensions. The largest deviations are for the thickest capsids and lie below the trend. This may reflect deviations from the thin shell scaling or local fluctuations in the coordination number that produce weak spots that dominate the response. Even the largest values of 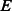 are an order of magnitude smaller than an fcc lattice with 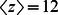 and the same interactions. It is interesting to ask whether the greater flexibility of all capsids and the variations in 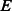 for specific capsids are important to function. This is outside the scope of the current paper, but one may speculate that viruses may be more rigid if they do not need to reform during their life cycle or are exposed to more extreme environments.

Experimental values of 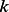 and 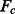 are only available for 5, viruses [3]. Our calculations reproduce the trends in these quantities and good quantitative agreement with experiments is obtained if the interaction strength is set to the value obtained from fits to protein stretching 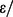
Å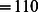 pN. Calculated values of 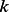 are consistently about a factor of 2 too small with this interaction strength. As discussed, the current coarse-grained model assumes that the separation between C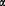 bonds determines the rate of change of forces. Better quantitative agreement could be obtained if the variation in force was related to the smaller separation between amino acids.

The variability in elastic properties of virus capsids has also been observed by Tama and Brooks [41]. They also considered only C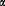 atoms and assigned Hookean springs between nearest-neighbors. They then analyzed the normal modes of the system and correlated them to structural changes of swelling rather than nanoindentation. It would be interesting to determine the frequency of normal modes that correspond most closely to indentation using this method. This would also enable rapid studies of the effect of capsid orientation on 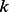, which has been found to be small in previous studies [16], [39], [40].

The studies presented here have focused on the experimentally accessible macroscopic response of capsids. Future studies should assess the variability in local response within capsids. Variations in local deformation may be correlated with changes in local coordination number and/or with the boundaries of proteins as in our earlier simulations and recent experiments on MVM [9]. It may also be possible to relate them to local magnitude variations of the eigenmodes obtained by normal mode analysis [41]. These studies could help explain the variations in 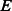 at a given 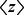 and will be a useful stepping stone towards modeling still larger biological systems.

## References

[1] Buenemann M, Lenz P (2007) Mechanical limits of viral capsids. Proc Natl Acad Sci USA 104: 9925–9930. 17545309 10.1073/pnas.0611472104 PMC1891220

[2] Buenemann M, Lenz P (2008) Elastic properties and mechanical stability of chiral and filled capsids. Phys Rev E 78: 051924. 10.1103/PhysRevE.78.051924 19113172

[3] Roos WH, Bruisma R, Wuite GJL (2010) Physical virology. Nature Physics 6: 733–743.

[4] Caspar D, Klug A (1962) Cold Spring Harbor Symposium on Quantitative Biology. 27: 1–24. 10.1101/sqb.1962.027.001.005 14019094

[5] Carrillo-Tripp M, Shepherd CM, Borelli IA, Venkataraman S, Lander G, et al (2009) VIPERdb2: and enhanced and web API enabled relational database for structural virology. Nucl Acids Res 37: D436–D442 Available: http://viperdb.scripps.edu/. 18981051 10.1093/nar/gkn840 PMC2686430

[6] Berman HM, Westbrook J, Feng Z, Gilliland G, Bhat TN, et al (2000) The Protein Data Bank. Nucl Acids Res 28: 235–242. 10592235 10.1093/nar/28.1.235 PMC102472

[7] Carrasco C, Carreira A, Schaap IAT, Serena PA, Gomez-Herrero J, et al (2006) DNA-mediated anisotropic mechanical reinforcement of a virus. Proc Natl Acad Sci USA 103: 13706–13711. 16945903 10.1073/pnas.0601881103 PMC1564217

[8] Carrasco C, Castellanos M, de Pablo PJ, Mateu MG (2008) Manipulation of the mechanical properties of a virus by protein engineering. Proc Natl Acad Sci USA 105: 4150–4155. 18334651 10.1073/pnas.0708017105 PMC2393779

[9] Castellanos M, Perez R, Carillo PJP, de Pablo PJ, Mateu MG (2012) Mechanical disassembly of single virus particles reveals kinetic intermediates predicted by theory. Bioph J 102: 2615–2624. 10.1016/j.bpj.2012.04.026 PMC3368135 22713577

[10] Michel JP, Ivanovska IL, Gibbons MM, Klug WS, Knobler CM, et al (2006) Nanoindentation studies of full and empty viral capsids and the effects of capsid protein mutations on elasticity and strength. Proc Natl Acad Sci USA 103: 6184–6189. 16606825 10.1073/pnas.0601744103 PMC1458852

[11] Klug WS, Bruinsma RF, Michel JP, Knobler CM, Ivanovska IL, et al (2006) Failure of viral shells. Phys Rev Lett 97: 228101. 17155845 10.1103/PhysRevLett.97.228101

[12] Baclayon M, Shoemaker GK, Uetrecht C, Crawford SE, Estes MK, et al (2011) Prestress strengthens the shell of Norwalk virus nanoparticles. Nano Lett 11: 4865–4869. 21967663 10.1021/nl202699r PMC4059365

[13] Arkhipov A, RoosWH, Wuite GJL, Schulten K (2009) Elucidating the mechanism behind irreversible deformation of viral capsids. Biophys J 97: 2061–2069. 19804738 10.1016/j.bpj.2009.07.039 PMC2756377

[14] Roos WH, Gibbons MM, Arkhipov A, Uetrecht C, Watts NR, et al (2010) Sqeezing protein shells: How continuum elastic models, molecular dynamics simulations and experiments coalesce at the 15 nanoscale. Biophys J 99: 1175–1181. 20713001 10.1016/j.bpj.2010.05.033 PMC2920642

[15] Roos WH, Gertsman I, May ER, Brooks III CL, Johnson JE, et al (2012) Mechanics of bacteriophage maturation. Proc Natl Acad Sci USA 109: 2342–2347. 22308333 10.1073/pnas.1109590109 PMC3289345

[16] Cieplak M, Robbins MO (2010) Nanoindentation of virus capsids in a molecular model. J Chem Phys 132: 015101. 20078182 10.1063/1.3276287

[17] Ding J, Smith AD, Geisler SC, Ma X, Arnold GF, et al (2002) Crystal structure of a human rhinovirus that displays part of the HIV-1 V3 loop and induces neutralizing antibodies against HIV-1. Structure 10: 999–1011. 12121655 10.1016/s0969-2126(02)00793-1

[18] Cieplak M, Hoang TX (2003) Universality classes in folding times of proteins Biophys J. 84: 475–488. 10.1016/S0006-3495(03)74867-X PMC1302628 12524300

[19] Cieplak M, Hoang TX, Robbins MO (2004) Thermal effects in stretching of Go-like models of titin and secondary structures. Proteins: Struct Funct Bio 56: 285–297. 10.1002/prot.20081 15211512

[20] Sułkowska JI, Cieplak M (2007) Mechanical stretching of proteins – a theoretical survey of the Protein Data Bank. J Phys: Cond Mat 19: 283201.

[21] Sułkowska JI, Cieplak M (2008) Selection of optimal variants of Go-like models of proteins through studies of stretching. Biophys J 95: 3174–3191. 18567634 10.1529/biophysj.107.127233 PMC2547460

[22] Sikora M, Sułkowska JI, Cieplak M (2008) Mechanical strength of 17 132 model proteins and cysteine slipknots. PloS Comp Biol 5: e1000547. 10.1371/journal.pcbi.1000547 PMC2759523 19876372

[23] Gibbons MM, Klug WS (2007) Nonlinear finite-element analysis of nanoindentation of viral capsids. Phys Rev E 75: 031901. 10.1103/PhysRevE.75.031901 17500720

[24] GibbonsMM KlugWS (2008) Influence of nonuniform geometry on nanoindentation of viral capsids. Biophys J 95: 3640–3649. 18621831 10.1529/biophysj.108.136176 PMC2553106

[25] Allen MP, Tildesley DJ (1987) “Computer simulations of liquids”. Oxford University Press, New York.

[26] Tsai J, Taylor R, Chothia C, Gerstein M (1999) The packing density in proteins: Standard radii and volumes. J Mol Biol 290: 253–266. 10388571 10.1006/jmbi.1999.2829

[27] Reddy VS, Natarajan P, Okerberg B, Li K, Damodaran KV, et al (2001) Virus Particle Explorer (VIPER), a website for virus capsid structures and their computational analyses. J Vir 75: 11943–11947. 10.1128/JVI.75.24.11943-11947.2001 PMC116089 11711584

[28] Vriend G, Verduin BJM, Hemminga MA (1986) Role of the N-terminal part of the coat protein in the assembly of cowpea chlorotic mottle virus. J Mol Biol 191: 453–460. 3820293 10.1016/0022-2836(86)90140-3

[29] Speir JA, Munshi S, Wang G, Baker TS, Johnson JE (1995) Structures of the native and swollen forms 16 of cowpea chlorotic mottle virus determined by X-ray crystallography and cro-electron microscopy. Structure 3: 63–78. 7743132 10.1016/s0969-2126(01)00135-6 PMC4191737

[30] Grest GS, Kremer K (1986) Molecular dynamics simulation for polymers in the presence of a heat bath. Phys Rev A 33: 3628–31. 10.1103/physreva.33.3628 9897103

[31] Veitshans T, Klimov D, Thirumalai D (1997) Protein folding kinetics: time scales, pathways, and energy landscapes in terms of sequence-dependent properties. Folding Des 2: 1–22. 10.1016/S1359-0278(97)00002-3 9080195

[32] Szymczak P, Cieplak M (2006) Stretching of proteins in a uniform flow. J Chem Phys 125: 164903. 17092135 10.1063/1.2358346

[33] Steele WA (1973) Physical interaction of gases with crystalline solids. 1. Gas-solid energies and properties of isolated adsorbed atoms. Surf Sci 36: 317–352.

[34] Zink M, Grubmueller H (2009) Mechanical properties of the icosahedral shell of southern bean mosaic virus: a molecular dynamics study. Biophys J 96: 1350–1363. 19217853 10.1016/j.bpj.2008.11.028 PMC2717248

[35] Feng S, Sen PN (1984) Percolation on elastic networks: new exponent and threshold. Phys Rev Lett 52: 217–219.

[36] Jacobs DJ, Thorpe MF (1995) Generic rigidity percolation: The pebble game. Phys Rev Lett 75: 4051–4. 10059802 10.1103/PhysRevLett.75.4051

[37] Mao X, Xu N, Lubensky TC (2010) Soft modes and elasticity of nearly isostatic lattices: Randomness and dissipation. Phys Rev Lett 104: 085504. 20366946 10.1103/PhysRevLett.104.085504

[38] Liu AJ, Nagel SR (2010) The jamming transition and the marginally jammed solids. Annu Rev Condens Matter Phys 1: 347–369.

[39] Arkhipov A, Freddolino PL, Schulten K (2006) Stability and dynamics of virus capsids described by coarse-grained modeling. Structure 14: 1767–1777. 17161367 10.1016/j.str.2006.10.003

[40] Arkhipov A, Wouter HR, Wuite GJL, Schulten K (2009) Elucidating the mechanism behind irreversible deformation of viral capsids. Bioph J 97: 2061–2069. 10.1016/j.bpj.2009.07.039 PMC2756377 19804738

[41] Tama F, Brooks III CL (2005) Diversity and identity of mechanical properties of icosahedral viral capsids studied with elastic network normal mode analysis. J Mol Biol 345: 299–314. 15571723 10.1016/j.jmb.2004.10.054

